# Prion Strain Differences in Accumulation of PrPSc on Neurons and Glia Are Associated with Similar Expression Profiles of Neuroinflammatory Genes: Comparison of Three Prion Strains

**DOI:** 10.1371/journal.ppat.1005551

**Published:** 2016-04-05

**Authors:** James A. Carroll, James F. Striebel, Alejandra Rangel, Tyson Woods, Katie Phillips, Karin E. Peterson, Brent Race, Bruce Chesebro

**Affiliations:** Laboratory of Persistent Viral Diseases, Rocky Mountain Laboratories, National Institute of Allergy and Infectious Disease, National Institutes of Health, Hamilton, Montana, United States of America; Creighton University, UNITED STATES

## Abstract

Misfolding and aggregation of host proteins are important features of the pathogenesis of neurodegenerative diseases including Alzheimer’s disease, Parkinson’s disease, frontotemporal dementia and prion diseases. In all these diseases, the misfolded protein increases in amount by a mechanism involving seeded polymerization. In prion diseases, host prion protein is misfolded to form a pathogenic protease-resistant form, PrPSc, which accumulates in neurons, astroglia and microglia in the CNS. Here using dual-staining immunohistochemistry, we compared the cell specificity of PrPSc accumulation at early preclinical times post-infection using three mouse scrapie strains that differ in brain regional pathology. PrPSc from each strain had a different pattern of cell specificity. Strain 22L was mainly associated with astroglia, whereas strain ME7 was mainly associated with neurons and neuropil. In thalamus and cortex, strain RML was similar to 22L, but in substantia nigra, RML was similar to ME7. Expression of 90 genes involved in neuroinflammation was studied quantitatively using mRNA from thalamus at preclinical times. Surprisingly, despite the cellular differences in PrPSc accumulation, the pattern of upregulated genes was similar for all three strains, and the small differences observed correlated with variations in the early disease tempo. Gene upregulation correlated with activation of both astroglia and microglia detected in early disease prior to vacuolar pathology or clinical signs. Interestingly, the profile of upregulated genes in scrapie differed markedly from that seen in two acute viral CNS diseases (LaCrosse virus and BE polytropic Friend retrovirus) that had reactive gliosis at levels similar to our prion-infected mice.

## Author Summary

Accumulation of aggregates of misfolded protein in brain is a common feature of the damage seen in several neurodegenerative diseases including prion disease, Alzheimer’s disease and Parkinson’s disease. In the present work three strains of prion disease differed in accumulation of the disease-associated prion protein (PrPSc) on neurons and astroglial cells. These patterns were first detectable in the thalamus at 40–60 days after inoculation. This coincided with initial detection of gliosis and PrPSc deposition, but was far in advance of clinical signs or spongiform pathology. In spite of the different patterns of cellular PrPSc deposition, these three strains had similar patterns of expression of a large number of genes known to be active during neuroinflammatory responses and gliosis. However, the gene upregulation in scrapie differed markedly from that seen in two neurovirulent viral diseases, which also had abundant glial responses similar to those observed with prion infection.

## Introduction

Several neurodegenerative diseases including Alzheimer’s disease (AD), Parkinson’s disease (PD), frontotemporal dementia (FTD) and prion diseases are characterized by accumulation of aggregates of misfolded protein in brain [1]. The particular protein or proteins involved in each of these diseases are different, but in each disease the protein misfolding appears to be spread within the brain by a seeding process where one misfolded aggregate can seed the misfolding of other normally folded molecules of the same protein by a mechanism known as “seeded polymerization” [2, 3]. In the case of prion diseases, seeded amplification results in increased levels of the misfolded protein and spread to adjacent brain regions. In addition, extracts from these brains can transmit prion disease to new individuals by experimental, iatrogenic or natural routes [4]. The realization that seeded polymerization is a similar process, not only in infectious prion diseases but also in some other non-infectious neurological diseases, has led to a resurgence of interest in studies of prion-like effects in many neurodegenerative diseases [5]. One goal is to develop common strategies of therapeutic intervention against the seeded polymerization events.

Prion diseases are slowly progressive, usually fatal brain diseases characterized by the development of vacuoles in the gray matter, prominent gliosis involving astroglia and microglia, and deposition in brain of aggregated partially protease-resistant isoforms (PrPSc or PrPres) derived from host-encoded normal prion protein (PrPC or PrPsen) [6]. These diseases occur naturally in humans and ruminants and can be transmitted to rodents, nonhuman primates, felines, mustelids and other animals. Interestingly, within a given animal species multiple strains of prion infectivity have been identified based primarily on differing patterns of regional brain pathology at the clinical end-point. The molecular explanation for the maintenance of diverse strain phenotypes in a single mouse strain with only one type of PrP protein sequence is not clear. However, the secondary structure of the PrPres aggregates is known to differ among certain strains, and such structures appear to be maintained during templated replication of prions using a single primary PrP protein sequence [7].

Most studies of prion strains have focused on strain-specific differences in the regional patterns of prion-induced vacuolar neuropathology and/or PrPSc deposition [8, 9, 10], but a few papers have also described strain differences in association of PrPSc with particular brain cell types. For example, at late clinical times sheep infected naturally or experimentally with sheep or mouse scrapie were found to have strain-specific patterns of PrPSc accumulation with neurons or glia [11–13]. In hamster experiments, accumulation of PrPSc in neuronal soma at the clinical end-point varied among 8 scrapie strains and appeared to correlate with shorter incubation periods [14]. In other studies using mice, morphological patterns of PrPSc deposition at the clinical end-point were shown to differ among certain scrapie strains; for example, ME7 was primarily neuronal, and 79A was both neuronal and astroglial [15]. Although certain patterns of cell association were clear in these experiments, the extensive spread and deposition of PrPSc at the clinical end-point might obscure the initial specificity of PrPSc for certain brain structures or cells. However, few preclinical studies have followed scrapie strain-specific PrPSc cellular associations. In two studies comparing scrapie strains ME7, 79A and 22L at 77 and 91 dpi after intra-hippocampal microinjection, strain-specific differences were seen in gliosis and synaptophysin staining, but cell specificities of PrPSc accumulations were not examined [16, 17].

Recently, after intracerebral microinjection of 22L scrapie in mice, we observed generation of new PrPSc beginning at 3–7 dpi, which was often on blood vessels near the injection site in the striatum [18]. In other microinjection experiments, we detected new PrPSc accumulations at 40 dpi in ipsilateral thalamus and cortex at levels 2–3 mm caudal to the injection site [19] suggesting that spread from the needle track to these sites was likely following neuronal circuitry. This strategy of microinjection followed by serial sectioning to identify the regions of interest at early times post-injection provides a useful approach to studying early events in the pathogenesis of scrapie infection in vivo. Although the intracerebral route is not a “natural” route, intracerebral infection does occur in humans via iatrogenic infection by contaminated surgical instruments and dural grafts. Thus studies involving this route may have practical relevance.

In the present study, dual-staining for PrPSc and for cell specific antigens was used to compare the cellular associations of PrPSc from three mouse-adapted scrapie strains at a time of early PrPSc deposition after microinjection in the striatum. To avoid artifacts, most studies were done at sites away from the needle track, such as thalamus, cortex and midbrain. In this work, PrPSc generated early after infection by the three strains tested was found to vary in its association with astroglia and neurons.

Because of the well-known association of micro- and astrogliosis with prion disease pathogenesis, we also studied whether prion strain-specific PrPSc deposition was correlated with the neuroinflammatory response seen during scrapie infection [20–25]. In the present paper expression of multiple neuroinflammatory genes in the thalamus was analyzed at preclinical time-points from 40 to 100 dpi using a sensitive real-time polymerase chain reaction (RT-PCR) method. Surprisingly, despite the differences seen in cell-association of PrPSc among the strains, all three strains had similar patterns of upregulation of genes associated with neuroinflammation. Interestingly, neuroinflammation early after prion infection in thalamus differed considerably from the pattern of genes upregulated in brains of mice infected with two acute CNS viruses that had similar levels of gliosis to the prion-infected mice.

## Results

### Differences in extent of early PrPSc generation after microinjection of 3 scrapie strains

**Fig 1:**
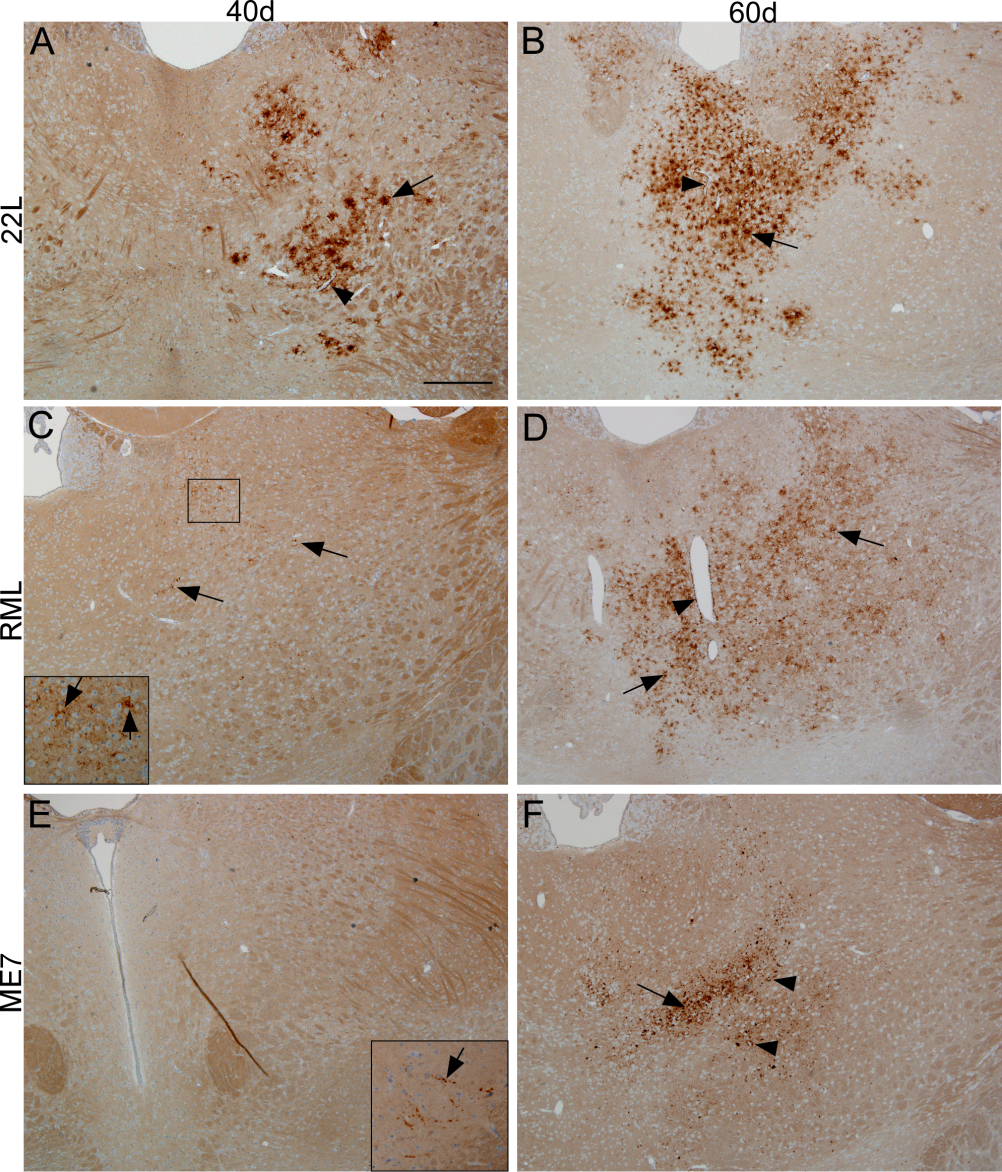
**Detection of PrPSc by immunohistochemistry using monoclonal antibody D13 in thalamus at 40 and 60 days post-infection by microinjection with scrapie strains 22L (A,B), RML (C,D), or ME7 (E,F).** (A) For strain 22L, at 40 dpi several areas of PrPSc deposition around apparent individual cells (arrow) and around blood vessels (arrowhead) were noted in dorsomedial (DM) region of the thalamus on the ipsilateral side. (B) At 60 dpi PrPSc was detected in the same areas but was more abundant than in (A). (C) After infection with RML, at 40 dpi small amounts of finely punctate PrPSc was barely visible (arrows) in clusters around large nuclei, but with magnification (inset) was easily seen in DM thalamus on ipsilateral side (arrows). (D) In contrast, at 60 dpi RML PrPSc deposits were widespread in DM thalamus (arrows) and were often on blood vessels (arrowhead), or in neuropil spaces between cells (zoom on figure to see details). (E) After ME7 infection, no PrPSc was seen in thalamus at 40 dpi, but a small amount of linear (axonal) punctate PrPSc was observed in the ipsilateral substantia nigra (inset, arrow). (F) At 60 dpi ME7 coarsely punctate PrPSc was noted in DM thalamus as amorphous aggregates of varying size (arrow). Occasional perineuronal PrPSc was also seen (arrowheads, zoom to see details). The area of ME7 PrPSc deposition was smaller than that for strains 22L and RML at this same time. Scale bar shown in panel A is 400 micrometers and applies to all panels.

In the current experiments we compared spread of PrPSc from three well-known scrapie strains (22L, RML and ME7) following microinjection of 0.5 μl of 10% scrapie brain homogenate into the striatum. In previous experiments, PrPSc was generated near the needle track site starting at 3–7 dpi and continued to spread in this local region [18]. This intracerebral microinjection caused a small amount of trauma to the local nerves and capillaries. However, PrPSc was subsequently found at 40 dpi in ipsilateral thalamus at a distance of 2–3 mm caudal to the injection site, suggesting that intracerebral microinjection did not alter long distance spread by neuronal circuitry [19]. The tempo of infection of the thalamus by the three different strains was determined by immunohistochemistry (IHC) analysis of PrPSc. At 40 dpi 22L PrPSc was readily detected in thalamus (Fig 1A), whereas RML and ME7 PrPSc were at the lower limit of detection (Fig 1C and 1E). In contrast, at 60 dpi PrPSc from all three strains was easily seen in thalamus, although ME7 was somewhat less abundant (Fig 1B, 1D and 1F). The faster pace of 22L infection may be due to the 10-25-fold higher amount of infectivity inoculated compared to RML and ME7 (see methods for details). In contrast, the titer of ME7 was 2-fold higher than that of RML, but still the onset of detectable PrPSc in thalamus was slower for ME7. Clinical disease was also delayed in ME7 (174 dpi for ME7 versus 149 dpi for RML), suggesting that this difference was a property of these two strains. The slower course of ME7 infection has been reported previously by others [10].

**Fig 2:**
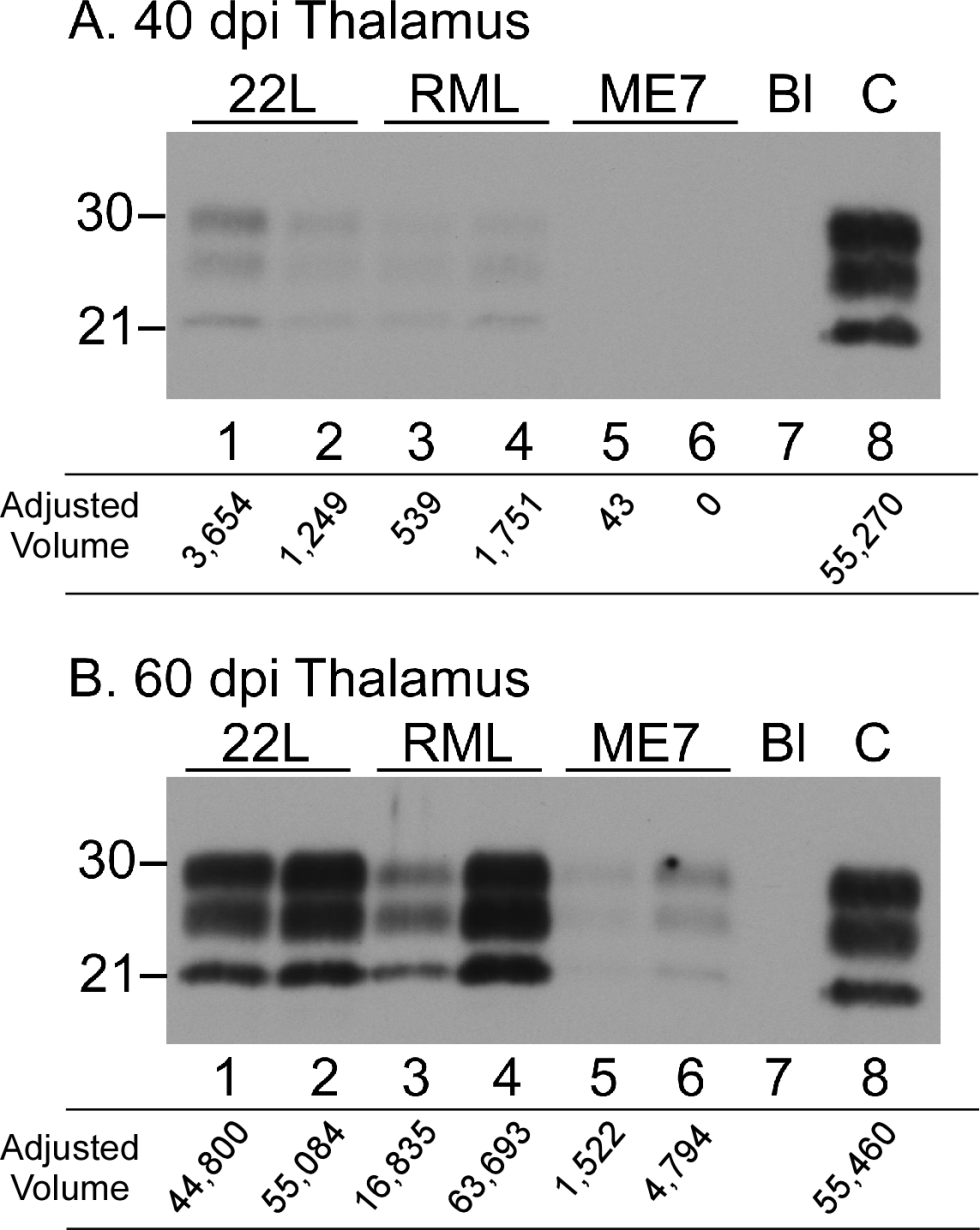
Western blots comparing PrPSc levels in thalamus at 40 and 60 dpi from three scrapie strains. (A) 40 dpi. (B) 60 dpi. On each western blot, two representative thalamus samples are shown for each scrapie strain. Scrapie strains shown are indicated across the top of each western blot. Lanes 1–6 were loaded with 0.36 mg of brain equivalents. In both panels, lane 7 (Bl) indicates an intentional blank lane, and lane 8 (C) is a positive control thalamus from a 60 dpi 22L sample loaded with 0.24 mg brain equivalents. Western blots were probed with anti-PrP antibody D13 and exposed to film for 8 minutes. To avoid issues with pixel saturation, densitometry was performed on a 4 minute exposure to film. The adjusted volumes (immunoreactive band volume with global background subtracted) in arbitrary units are given below each lane. At both time points shown the mice inoculated with scrapie strain ME7 had significantly less detectable PrPSc (lanes 5&6) than was seen in samples from 22L- and RML-infected mice.

To obtain a quantitative analysis of thalamic PrPSc levels in this experiment, thalamic brain tissue of replicate mice was analyzed for PrPSc by immunoblot and densitometry. In thalamic homogenates of mice infected with strains 22L or RML PrPSc bands were similar but weak at 40 dpi, and were much more intense at 60dpi (Fig 2). In contrast, in samples from ME7-infected mice PrPSc bands were undetectable at 40dpi, but weak bands were visible 60dpi. In fact, the band intensities observed in ME7 samples from 60dpi closely resembled that seen in 22L and RML samples at 40dpi (compare adjusted volumes for Fig 2B ME7 to 2A 22L and RML).

### Strain differences in cell-type association of PrPSc in thalamus

**Fig 3:**
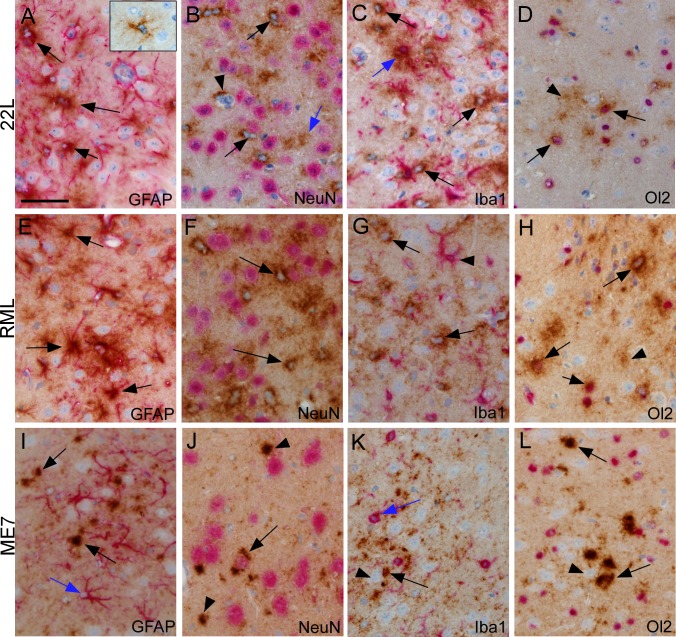
**Cell-type associations of PrPSc in thalamus after microinjection with scrapie strains 22L, 40 dpi (A-D), RML, 60 dpi (E-H) and ME7, 60 dpi (I-L)**. PrPSc was detected by IHC with monoclonal antibody D13, and slides were also dual-stained with a second antibody to detect neurons (NeuN), astroglia (GFAP), microglia (Iba1) and oligodendroglia (Ol2), as described in methods. (A) Several cells positive for both 22L PrPSc and GFAP (arrows) are noted. Inset shows a PrPSc-positive stellate cell at 20 dpi that was GFAP-negative. (B) 22L PrPSc-positive cells negative for Neu-N (arrows) are shown. Arrowhead shows PrPSc on a small blood vessel. Blue arrow shows PrPSc in neuropil not associated with any cell body. (C) One cell with 22L PrPSc and Iba1 co-association is shown (blue arrow). Several PrPSc-positive cells negative for Iba1 are also seen (black arrows). (D) Cells positive for 22L PrPSc and Olig2 (Ol2) (arrows) plus neuropil PrPSc (arrowhead) is seen. (E) RML PrPSc co-associates with GFAP-positive astroglia (arrows). (F) Intense RML PrPSc staining (arrows) is seen around Neu-N-negative cells with pale blue nuclei. Diffuse PrPSc staining is seen in neuropil background and is often adjacent to Neu-N-positive neurons, therefore possible association with neurons is difficult to exclude. (G) Lack of co-localization of RML PrPSc and Iba1. PrPSc-positive cells (arrows) are distinguishable from Iba1-positive cells (arrowhead). (H) Several cells are co-stained for RML PrPSc and Olig2 (arrows). Less dense PrPSc covers much of background neuropil. (I) After ME7 infection shows GFAP-positive astrocytes near larger PrPSc deposits (black arrows) in neuropil, but most astrocytes are PrPSc-negative (blue arrow). (J) Neu-N-positive neuron with ME7 PrPSc co-localization (arrow) on ipsilateral side. Additional PrPSc staining in larger aggregates (arrowheads) appears in neuropil. (K) No accumulation of ME7 PrPSc on Iba1-positive cells (blue arrow). PrPSc was seen in neuropil (black arrow) and possibly associated with large unstained neurons (arrowhead). (L) Large ME7 PrPSc aggregates in neuropil (arrows) with no evidence for accumulation on cells positive for Olig2 (arrowhead). Scale bar shown in panel A is 50 μm and applies to all panels.

Thalamus was an area with reliable early PrPSc generation, which was also sufficiently far (2–3 mm) from the needle track in the striatum to avoid artifacts due to the needle track wound. Therefore, we studied thalamus in mice at various times after microinjection of scrapie agent from strains 22L, RML or ME7 by dual-staining IHC to detect the early cell associations of newly generated PrPSc. Sections were examined at several different times from 20 to 100dpi, and the time-points giving the optimal density of PrPSc for evaluation of the cell type associations are shown in Fig 3. At 20 dpi PrPSc from strain 22L was associated with stellate cells, which did not stain positive for GFAP (Fig 3A, inset). Based on their morphology and staining for S100, these cells were likely astroglia that were not yet fully reactive and thus did not express detectable levels of GFAP [26]. At 40 dpi we also observed cells with a star-like morphology expressing PrPSc, but these cells expressed GFAP and thus appeared to be astroglia (Fig 3A). Surprisingly, at 40 dpi using anti-NeuN neuronal staining, 22L PrPSc was not associated with NeuN-positive neurons, but instead was associated with NeuN-negative cells with smaller nuclei that were consistent with astroglia (Fig 3B). Less dense PrPSc was also seen in the neuropil between various cell bodies, but this material could not be assigned to any particular cell type since processes of many cell types occupy this space (Fig 3B, blue arrow). After dual staining with anti-Iba1 to detect microglia, 22L PrPSc was also occasionally associated with microglia (Fig 3C, blue arrow), but several PrPSc-positive cells negative for Iba1 staining are also seen (black arrows). Dual-staining with anti-Olig2, typically a marker for oligodendroglia, also showed co-association with 22L PrPSc (Fig 3D), and therefore these cells were likely to be Olig2-positive reactive astroglia, which have been described previously [27].

Similar examination of strain RML PrPSc in thalamus with these same four dual stains showed a pattern nearly identical to that seen with 22L PrPSc (Fig 3E–3H). RML was examined at 60 dpi in thalamus because the extent of PrPSc spread was optimal for cell-type analysis at that time and was very similar in density to 22L at 40 dpi. Thus, in thalamus 22L and RML PrPSc seemed to be mainly associated with astroglia, but were also associated to a lesser extent with microglia and oligodendroglia. These data indicated that astroglia were either a primary target of infection and PrPSc generation by these two strains or that astroglia were efficient at sequestering 22L and RML PrPSc made by other cell types.

In contrast to above data, results with strain ME7 in thalamus at 60 dpi were quite different. There was no obvious association of ME7 PrPSc with GFAP, Iba1 or Olig2, indicating this PrPSc was not associated with astroglia, microglia or oligodendroglia (Fig 3I, 3K, and 3L). In contrast, ME7 PrPSc was associated with NeuN-positive neurons (Fig 3J), and also appeared to be present in numerous aggregates of varying size located in the neuropil (Fig 3I–3L). Thus neurons and neuropil seemed to be the main sites of deposition of ME7 PrPSc in thalamus.

### Strain differences in cell association of PrPSc in other brain regions

**Fig 4:**
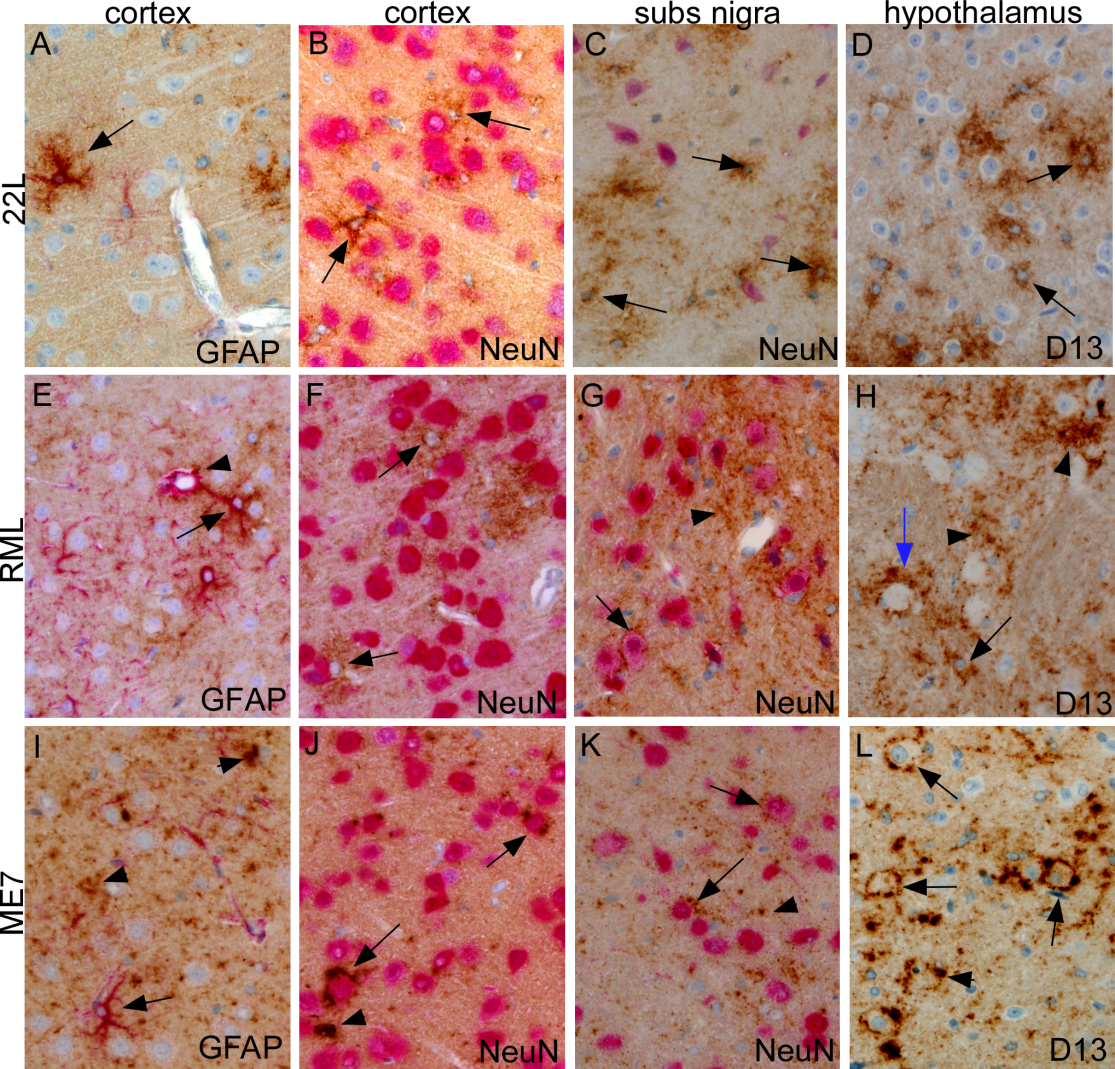
**Cell-type associations of PrPSc in cortex, substantia nigra and hypothalamus after striatal microinjection of scrapie strains 22L (A-D), RML (E-H) or ME7 (I-L).** PrPSc was detected by IHC with monoclonal antibody D13 (brown color), and some slides were also dual-stained with a second antibody to detect neurons (NeuN) or astroglia (GFAP) as described in methods. (A) At 40 dpi 22L PrPSc is associated with a stellate GFAP-positive astrocyte (arrow). Nearby larger neurons and vascular endothelial cells are PrPSc-negative. (B) At 40 dpi stellate cells (arrows), likely to be astrocytes, are positive for 22L PrPSc and are intermingled with neurons stained with anti-NeuN (pink). (C) At 40 dpi in substantia nigra 22L PrPSc is not associated with Neu-N-positive neurons (pink) but instead surrounds cells with small blue nuclei, possibly astrocytes. (D) In hypothalamus at 60 dpi single stained 22L PrPSc surrounds small cells with blue nuclei (arrows) and is less often adjacent to round neurons with prominent nucleoli. (E) At 60 dpi RML PrPSc is associated with a GFAP-positive astrocyte (arrow) and also with perivascular astrocyte surrounding a small blood vessel (arrowhead). (F) In cortex at 60 dpi RML PrPSc surrounds cells with small nuclei (arrows) near Neu-N-positive neurons, similar to 22L in panel (B). (G) In substantia nigra at 60 dpi RML PrPSc is associated with cell surface of two Neu-N-positive neurons (arrows). PrPSc is also seen in the neuropil between neurons (arrowhead). (H) At 80 dpi RML PrPSc in hypothalamus, detected by single staining with D13, is associated with neuropil (arrowheads), blood vessel (blue arrow) and probable astroglia with small nuclei (black arrow). (I) At 100 dpi ME7 PrPSc in cortex is deposited in fine and coarse aggregates in neuropil (arrowheads), but is not associated with GFAP-positive astroglia (arrow). (J) At 100 dpi in cortex ME7 PrPSc is associated with NeuN-positive neurons (arrows). Occasional coarse aggregates are also seen in the neuropil (arrowhead). (K) At 60 dpi in substantia nigra ME7 PrPSc surrounds some Neu-N-positive neurons and also is seen as punctate deposits (arrowheads) near neurons and as linear staining suggestive of axons. (L) At 100 dpi in hypothalamus ME7 PrPSc is shown using single staining in a perineuronal distribution (arrows) and also as coarse neuropil aggregates (arrowheads). Scale bar shown in panel A is 50 μm and applies to all panels.

PrPSc from these three scrapie strains is known to be deposited in other brain regions in addition to thalamus. Therefore, we also examined the cell-type association of PrPSc in several other brain regions, including cerebral cortex, substantia nigra (sn) and hypothalamus (ht), which all showed evidence of detectable PrPSc at preclinical times just slightly later than in thalamus. In cortex, 22L PrPSc was associated with GFAP-positive stellate cells (Fig 4A) that appeared to be astroglia and were not NeuN-positive neurons (Fig 4B). In sn and ht, 22L PrPSc was also associated with similar NeuN-negative astroglia with a stellate morphology (Fig 4C and 4D).

In cortex RML PrPSc was also associated with astroglia (Fig 4E and 4F), but surprisingly, in sn and ht, RML PrPSc was associated with neurons and neuropil (Fig 4G and 4H). The reasons for this regional difference in cellular associations of RML PrPSc were not clear.

Similar to the situation in thalamus, ME7 PrPSc was associated with neurons and neuropil in cortex, sn and ht. ME7 PrPSc was not associated with GFAP-positive cells (Fig 4I), but there was abundant PrPSc deposition in neuropil, association with NeuN-positive neurons in cortex (Fig 4J) and in sn (Fig 4K). Similarly, in hypothalamus, ME7 PrPSc deposits were noted surrounding numerous neurons and were also in the neuropil (Fig 4L).

In summary, at relatively early stages of scrapie infection in several brain regions, strain 22L PrPSc showed a consistent association with astroglia and occasional association with microglia, whereas strain ME7 PrPSc was associated with neurons and neuropil. In contrast, strain RML PrPSc was associated mainly with astroglia in thalamus and cortex, but with neurons and neuropil in sn and ht.

### Scrapie strain differences in expression of gliosis markers in thalamus at preclinical times after infection

**Fig 5:**
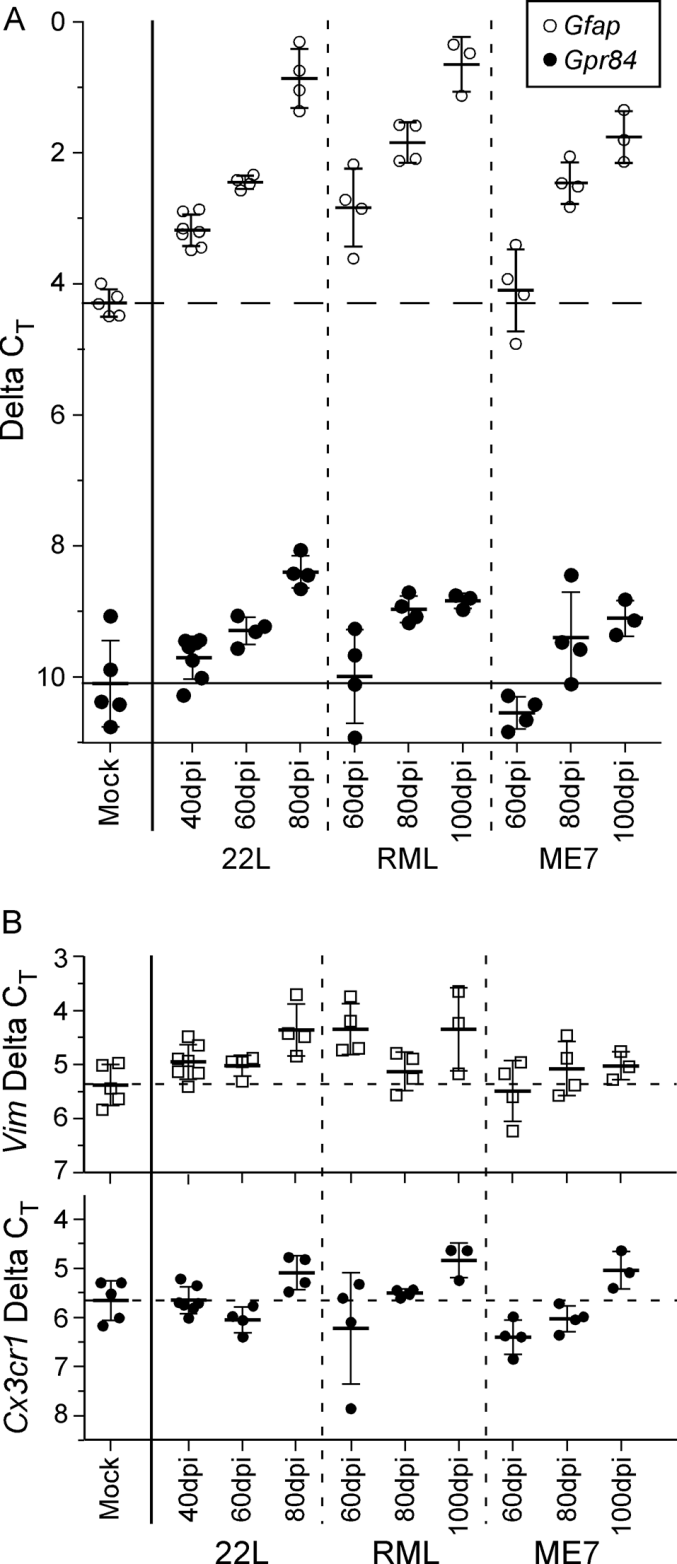
Transcript analysis of astroglial and microglial markers in thalamic RNA samples from mice infected with scrapie strains 22L, RML, or ME7. (A) The ΔC_T_ of *Gfap*, an indicator of astroglial activation (open circles), showed astrogliosis was significantly increased early in 22L-infected thalamus (40 dpi) and increased further at 60 and 80 dpi. Infection with RML reached a similar increase in *Gfap* by 60 dpi, whereas ME7-infected mice demonstrated a significant increase in *Gfap* similar to that of RML and 22L by 80 dpi. Likewise, the ΔC_T_ of *Gpr84*, an indicator of microglial activation (closed circles), illustrated a temporal increase in thalamic expression during infection by all three scrapie strains tested, albeit delayed by 20 dpi relative to *Gfap*. The dashed horizontal line indicates the mean ΔC_T_ of *Gfap* and the solid horizontal line indicates the mean *Gpr84* ΔC_T_ in mock infected mice. (B) The relatively unchanged expression of vimentin (*Vim*), an astrocyte associated gene, or the chemokine receptor *Cx3cr1*, a microglia associated gene, suggested the early transcriptional changes observed in the gliosis markers *Gfap* and *Gpr84* in infected thalamus were not due to an overt expansion of either cell population. The dashed horizontal lines indicate the mean ΔC_T_ in mock infected mice as a reference. The bars for all samples indicate the mean and 1 standard deviation. The ΔC_T_ for each mouse RNA sample was calculated by subtracting the geometric mean C_T_ of three housekeeping genes (*Actin*, *Gapdh*, and *Hsp90ab1*) from the C_T_ of the gene of interest. Each dot represents an individual mouse.

We next carried out experiments to investigate the functional biochemical effects of scrapie infection in thalamus at early times post-infection using these same three scrapie strains. Because of the strong association of PrPSc with glia and the early appearance of gliosis prior to visible neuronal vacuolation or cell death, we focused on analysis of transcript levels of a group of genes previously thought to be involved in neuroinflammation and gliosis [20]. Initially we quantified the amount of detectable gliosis in the thalamus of preclinical scrapie-infected mice, using *Gfap* and *Vimentin* as markers of astroglia, and *Gpr84 and Cx3cr1*, as markers of microglia (Fig 5A and 5B) [28–30]. For scrapie strains 22L and RML, an increase in *Gfap* expression compared to mock-infected mice was observed at the earliest times of significant detection of PrPSc in thalamus by IHC (40 and 60 dpi, respectively), and further increases were also seen at two subsequent time-points 20 and 40 days later (Fig 5). For strain ME7, which was slightly slower than RML in PrPSc generation, *Gfap* upregulation was not seen until 80dpi (Fig 5). Interestingly, significant expression of the microglial activation marker, *Gpr84*, in the thalamus was delayed by 20 dpi relative to that of *Gfap* for all three strains; however, *Gpr84* levels increased for all strains at later time-points (Fig 5). For all three prion strains *Vimentin* and *Cx3cr* did not increase at these same early time-points of infection, suggesting that these genes were not markers of glial activation in these prion models.

### Comparison of transcript levels for various cytokine and chemokine receptor and ligand genes after infection with scrapie strains

**Fig 6:**
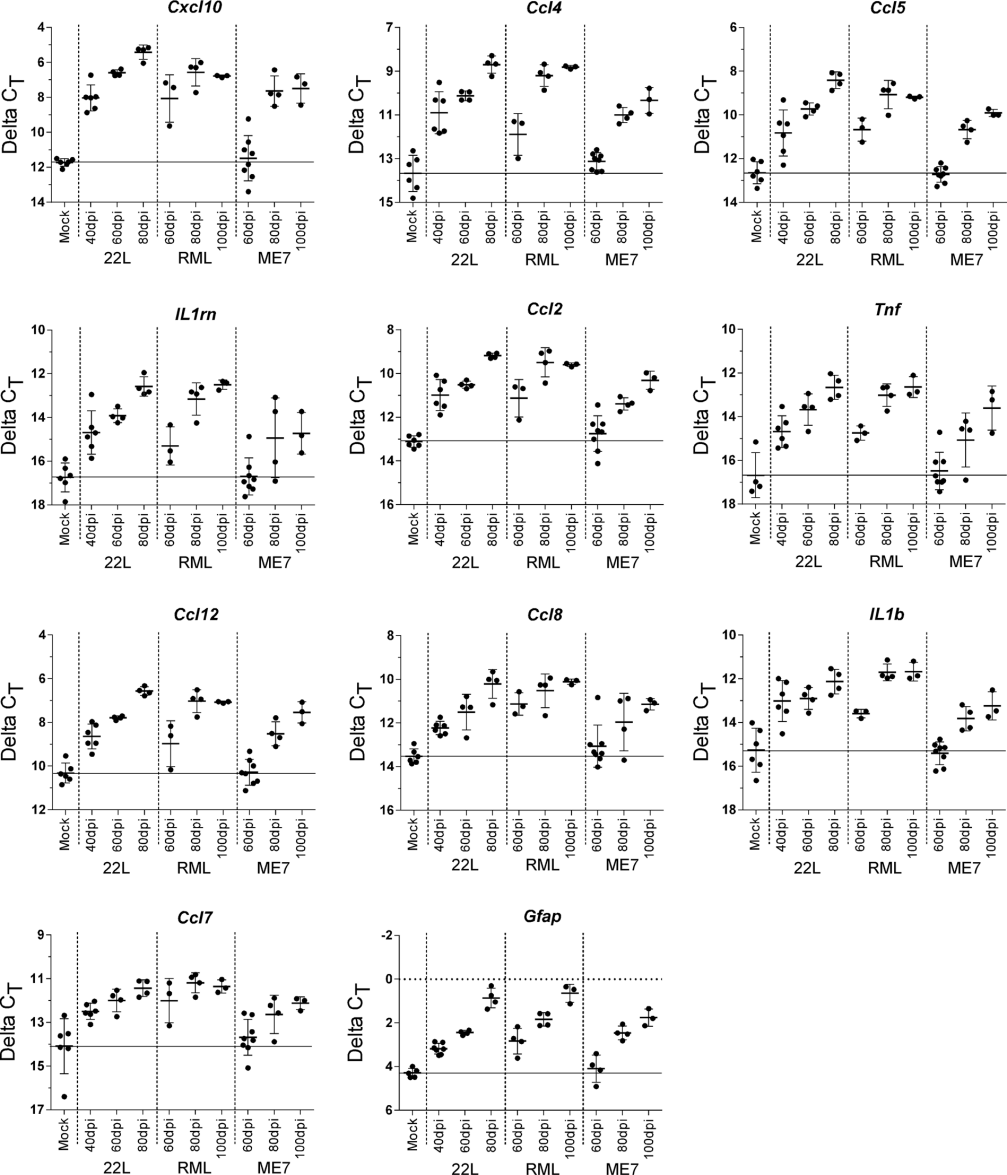
Inflammatory genes significantly increased in the thalamus of scrapie-infected mice. The changes in transcription for 86 inflammatory cytokines and their receptors was analyzed by qRT-PCR and categorized by time of increase (S1 Table for complete list of genes tested). The 11 genes shown here demonstrated a significant increase in expression in either 22L-infected or RML-infected thalamus of mice at the earliest time assessed (40 dpi and 60 dpi, respectively). The solid horizontal line on each graph indicates the mean ΔC_T_ in mock infected mice as a reference. The bars for all samples indicate the mean and 1 standard deviation. The ΔC_T_ for each mouse RNA sample was calculated by subtracting the geometric mean C_T_ of three housekeeping genes (*Actin*, *Gapdh*, and *Hsp90ab1*) from the C_T_ of the gene of interest. Each dot represents an individual mouse.

Having established that astroglial and microglial activation markers were detectable early in the infected thalamus, we used qRT-PCR to analyze expression levels of 86 genes known to be associated with neuroinflammation (S1 Table). Thalamic mRNA obtained at three preclinical times from mice infected with strains 22L, RML, or ME7 was studied. Eleven genes were found to be significantly upregulated after 22L or RML infection at the earliest times measured (40 dpi for 22L and 60 dpi for RML) (Fig 6). These genes included the astrogliosis marker, *Gfap*, as well as other genes likely to be expressed by astroglia, including *Cxcl10* and *Ccl2*. However, other genes in this group, such as *Tnf*, are thought to be mainly expressed by microglia. The remaining genes could have been expressed by astroglia, microglia or other cell types such as neurons or oligodendroglia. At later time-points more genes were upregulated after infection with each of the scrapie strains. Table 1 shows fold change and statistical significance at 40, 60 and 80 dpi of a list of 32 genes found to be upregulated at least 2.0 fold after infection with 22L at 80 dpi, and genes higher than 3.0 fold change were highlighted in black. Tables 2 and 3 show the fold change for these same genes at 60, 80 and 100 dpi after RML or ME7 respectively. With each strain there was a progressive increase in the number of upregulated genes over time. RML appeared to be about 20 days slower than 22L, but using 3.0 fold change as a cutoff, there were five genes upregulated after RML, but not after 22L infection (*Cx3cr1*, *Il4*, *Cxcl5*, *Ccr6*, and *Ccl11*). ME7 was more than 20 days slower than RML, and at 100 dpi only 14 genes were elevated above the 3.0 fold change cutoff (Table 3)

**Table 1:** Quantitative analysis by qRT-PCR of fold change of mRNA for specific genes in thalamus of mice microinjected with scrapie strain 22L at 40, 60 and 80 days post-injection.

	22L 40dpi (n = 8)^a^	22L 60dpi (n = 4)	22L 80dpi (n = 4)	
Gene^b^	FC^c^	FC	FC	Description
*Cxcl10*	**12.9** **	**35.0** ***	**78.9** ***	Chemokine (C-X-C motif) ligand 10
*Ccl4*	**6.9** **	**11.7** ***	**31.5** ***	Chemokine (C-C motif) ligand 4
*IL12b*	2.2	**11.6** *	**30.6** ***	Interleukin 12 p40
*Cxcl9*	3.2	**5.4** *	**26.4** ***	Chemokine (C-X-C motif) ligand 9
*Cxcl13*	1.2	**5.0** ***	**23.9** ***	Chemokine (C-X-C motif) ligand 13
*Ccl5*	**3.5** *	**7.5** ***	**18.7** ***	Chemokine (C-C motif) ligand 5
*Il1rn*	**4.2** *	**7.1** ***	**17.8** ***	Interleukin 1 receptor antagonist
*Ccl2*	**4.4** **	**6.1** ***	**15.3** ***	Chemokine (C-C motif) ligand 2
*Tnf*	**3.7** *	**7.5** **	**15.2** ***	Tumor necrosis factor
*Ccl12*	**3.2** **	**5.8** ***	**13.4** ***	Chemokine (C-C motif) ligand 12
*Gfap*	2.3***	**3.6** ***	**10.9** ***	Glial fibrillary acidic protein
*Ccl8*	2.5***	**4.1** **	**9.9** ***	Chemokine (C-C motif) ligand 8
*Il1b*	**4.7** **	**5.1** ***	**8.8** ***	Interleukin 1 beta
*Ccl3*	2.4*	**3.0** ***	**7.9** ***	Chemokine (C-C motif) ligand 3
*Ccl9*	2.4**	**3.5** ***	**6.5** ***	Chemokine (C-C motif) ligand 9
*Ccl6*	2.6***	**4.2** ***	**6.3** ***	Chemokine (C-C motif) ligand 6
*Ccl7*	**3.0** **	**4.3** **	**6.3** ***	Chemokine (C-C motif) ligand 7
*Osm*	1.5	**3.6** *	**6.1** **	Oncostatin M
*Il27*	1.5	1.2	**5.4** *	Interleukin 27
*Il1a*	1.8**	2.5***	**4.2** ***	Interleukin 1 alpha
*Il2rg*	1.2	1.6**	**3.5** ***	Interleukin 2 receptor, gamma chain
*Aif1*	1.4	1.5	**3.4** **	Allograft Inflammatory Factor 1
*Gpr84*	1.3	1.8	**3.3** ***	G protein coupled receptor 84
*Ccr5*	2.8	2.7	3.3	Chemokine (C-C motif) receptor 5
*Ccr3*	1.3	2.9***	**3.0** ***	Chemokine (C-C motif) receptor 3
*Il2rb*	-2.4	1.9	**3.0** ***	Interleukin 2 receptor, beta chain
*Cxcr3*	1.1	1.9	2.9*	Chemokine (C-X-C motif) receptor 3
*Il4*	1.2	1.8	2.8	Interleukin 4
*Ccr1*	1.5	1.6*	2.8***	Chemokine (C-C motif) receptor 1
*Il5*	-1.0	2.3*	2.6*	Interleukin 5
*Cxcl5*	1.0	2.6*	2.4**	Chemokine (C-X-C motif) ligand 5
*Ccr6*	-1.1	1.9	2.3	Chemokine (C-C motif) receptor 6
*Cxcl1*	2.0	**3.7** ***	2.3	Chemokine (C-X-C motif) ligand 1
*Ccl11*	1.2	1.8	2.3**	Chemokine (C-C motif) ligand 11
*Il10ra*	1.0	1.3*	2.2***	Interleukin 10 receptor, alpha

^a^ the number of mice per experimental condition.

^b^ Genes shown were found to show a greater than 2.0 fold change at 80 dpi after 22L infection out of 86 neuroinflammatory genes analyzed (S1 Table). Larger bolded numerals highlight genes having a fold change of 3.0 or greater and a P value of <0.05.

^c^ Fold change (FC) relative to mock controls (n = 6). P values are calculated using the Student’s t-test for comparison of the 2^(- Delta Ct)^ values for each gene in control mice versus infected mice.

* P value ≤ 0.05

** P value ≤ 0.01

*** P value ≤ 0.001

**Table 2:** Quantitative analysis by qRT-PCR of fold change of mRNA for specific genes in thalamus of mice microinjected with scrapie strain RML at 60, 80 and 100 days post-injection.

	RML 60dpi (n = 3)^a^	RML 80dpi (n = 4)	RML 100dpi (n = 3)	
Gene^b^	FC^c^	FC	FC	Description
*Cxcl10*	**12.6** **	**35.7** ***	**30.7** ***	Chemokine (C-X-C motif) ligand 10
*Ccl4*	**3.5** **	**22.2** ***	**29.1** ***	Chemokine (C-C motif) ligand 4
*IL12b*	3.4	**12.4** ***	**13.5** ***	Interleukin 12 p40
*Cxcl9*	1.5	**12.5** **	**9.6** **	Chemokine (C-X-C motif) ligand 9
*Cxcl13*	1.7	**7.2** ***	**10.9** ***	Chemokine (C-X-C motif) ligand 13
*Ccl5*	**3.9** ***	**11.9** ***	**10.9** ***	Chemokine (C-C motif) ligand 5
*Il1rn*	2.7*	**12.1** ***	**19.0** ***	Interleukin 1 receptor antagonist
*Ccl2*	**3.9** **	**12.3** ***	**11.4** ***	Chemokine (C-C motif) ligand 2
*Tnf*	**3.6** *	**11.8** ***	**15.3** ***	Tumor necrosis factor
*Ccl12*	2.5*	**9.8** ***	**9.5** ***	Chemokine (C-C motif) ligand 12
*Gfap*	2.8**	**5.5** ***	**12.7** ***	Glial fibrillary acidic protein
*Ccl8*	**5.3** ***	**8.0** ***	**10.7** ***	Chemokine (C-C motif) ligand 8
*Il1b*	**3.2** **	**11.8** ***	**12.1** ***	Interleukin 1 beta
*Ccl3*	1.2	**5.9** ***	**7.9** ***	Chemokine (C-C motif) ligand 3
*Ccl9*	1.4	**3.8** ***	**5.5** ***	Chemokine (C-C motif) ligand 9
*Ccl6*	1.7*	**4.9** ***	**8.1** ***	Chemokine (C-C motif) ligand 6
*Ccl7*	**4.2** *	**7.4** ***	**6.6** ***	Chemokine (C-C motif) ligand 7
*Osm*	1.5	**3.4** *	**4.2** *	Oncostatin M
*Il27*	1.7	**4.7** *	3.1	Interleukin 27
*Il1a*	1.5*	**3.5** ***	**3.6** ***	Interleukin 1 alpha
*Il2rg*	1.2	2.5**	**3.0** ***	Interleukin 2 receptor, gamma chain
*Aif1*	2.5	1.3	**3.6** ***	Allograft Inflammatory Factor 1
*Gpr84*	1.1	2.2**	2.4***	G protein coupled receptor 84
*Ccr5*	1.5	3.0	2.6	Chemokine (C-C motif) receptor 5
*Ccr3*	1.1	2.8***	2.1***	Chemokine (C-C motif) receptor 3
*Il2rb*	1.5	1.6	2.5**	Interleukin 2 receptor, beta chain
*Cxcr3*	1.5	**3.2** **	1.8	Chemokine (C-X-C motif) receptor 3
*Il4*	-1.2	**4.5** *	**3.2** *	Interleukin 4
*Ccr1*	1.2	2.4**	2.7***	Chemokine (C-C motif) receptor 1
*Il5*	2.3**	2.3*	1.2	Interleukin 5
*Cxcl5*	-1.3	**5.0** ***	**4.8** ***	Chemokine (C-X-C motif) ligand 5
*Ccr6*	2.2	2.2	**4.6** *	Chemokine (C-C motif) receptor 6
*Cxcl1*	1.0	**3.5** **	**3.5** *	Chemokine (C-X-C motif) ligand 1
*Ccl11*	1.4	**3.2** **	**3.4** ***	Chemokine (C-C motif) ligand 11
*Il10ra*	-1.2	1.4**	1.6**	Interleukin 10 receptor, alpha

^a^ the number of mice per experimental condition.

^b^ Genes shown were found to show a greater than 2.0 fold change at 80 dpi after 22L infection out of 86 neuroinflammatory genes analyzed (S1 Table). Larger bolded numerals highlight genes having a fold change of 3.0 or greater and a P value of <0.05.

^c^ Fold change (FC) relative to mock controls (n = 6). P values are calculated using the Student’s t-test for comparison of the 2^(- Delta Ct)^ values for each gene in control mice versus infected mice.

* P value ≤ 0.05

** P value ≤ 0.01

*** P value ≤ 0.001

**Table 3:** Quantitative analysis by qRT-PCR of fold change of mRNA for specific genes in thalamus of mice microinjected with scrapie strain ME7 at 60, 80 and 100 days post-injection.

	ME7 60dpi (n = 8^a^)	ME7 80dpi (n = 4)	ME7 100dpi (n = 3)	
Gene^b^	FC^c^	FC	FC	Description
*Cxcl10*	1.2	**17.0** **	**18.8** ***	Chemokine (C-X-C motif) ligand 10
*Ccl4*	1.5	**6.4** ***	**10.1** ***	Chemokine (C-C motif) ligand 4
*Il12b*	1.1	2.5	**4.1** **	Interleukin 12 p40
*Cxcl9*	-1.1	1.3	4.3*	Chemokine (C-X-C motif) ligand 9
*Cxcl13*	-1.6	-1.1	6.1*	Chemokine (C-X-C motif) ligand 13
*Ccl5*	-1.1	**3.9** ***	**6.7** ***	Chemokine (C-C motif) ligand 5
*Il1rn*	1.0	3.5	4.1*	Interleukin 1 receptor antagonist
*Ccl2*	1.3	**3.3** ***	**7.0** ***	Chemokine (C-C motif) ligand 2
*Tnf*	1.1	2.9	**7.9** **	Tumor necrosis factor
*Ccl12*	1.0	**3.5** **	**6.8** ***	Chemokine (C-C motif) ligand 12
*Gfap*	1.2	**3.6** ***	**5.9** ***	Glial fibrillary acidic protein
*Ccl8*	1.4	**3.0** *	**5.2** ***	Chemokine (C-C motif) ligand 8
*Il1b*	-1.1	2.7*	**4.1** **	Interleukin 1 beta
*Ccl3*	-1.3	1.9**	2.8***	Chemokine (C-C motif) ligand 3
*Ccl9*	-1.9**	1.6	2.2**	Chemokine (C-C motif) ligand 9
*Ccl6*	1.3	2.6***	**3.1** ***	Chemokine (C-C motif) ligand 6
*Ccl7*	1.3	2.7*	**3.9** **	Chemokine (C-C motif) ligand 7
*Osm*	1.8	1.1	2.7	Oncostatin M
*Il27*	-1.2	2.6	4.3	Interleukin 27
*Il1a*	1.3*	2.0*	2.4***	Interleukin 1 alpha
*Il2rg*	-1.4*	-1.0	1.3	Interleukin 2 receptor, gamma chain
*Aif1*	-1.1	2.9	2.0**	Allograft Inflammatory Factor 1
*Gpr84*	-1.4	1.6	2.0*	G protein coupled receptor 84
*Ccr5*	1.4*	1.2	2.1	Chemokine (C-C motif) receptor 5
*Ccr3*	1.4*	1.3	1.6**	Chemokine (C-C motif) receptor 3
*Il2rb*	1.1	1.3	2.2*	Interleukin 2 receptor, beta chain
*Cxcr3*	-1.4	-1.9	1.3	Chemokine (C-X-C motif) receptor 3
*Il4*	1.7	-1.3	2.0	Interleukin 4
*Ccr1*	1.0	-1.0	1.7	Chemokine (C-C motif) receptor 1
*Il5*	-1.2	-1.8	1.5	Interleukin 5
*Cxcl5*	1.5	**4.1** ***	2.5*	Chemokine (C-X-C motif) ligand 5
*Ccr6*	1.4	3.3	3.7	Chemokine (C-C motif) receptor 6
*Cxcl1*	1.9*	1.6	2.4	Chemokine (C-X-C motif) ligand 1
*Ccl11*	1.4	-1.3	1.7	Chemokine (C-C motif) ligand 11
*Il10ra*	-1.2	-1.1	1.3*	Interleukin 10 receptor, alpha

^a^ the number of mice per experimental condition.

^b^ Genes shown were found to show a greater than 2.0 fold change at 80 dpi after 22L infection out of 86 neuroinflammatory genes analyzed (S1 Table). Larger bolded numerals highlight genes having a fold change of 3.0 or greater and a P value of <0.05.

^c^ Fold change (FC) relative to mock controls (n = 6). P values are calculated using the Student’s t-test for comparison of the 2^(- Delta Ct)^ values for each gene in control mice versus infected mice.

* P value ≤ 0.05

** P value ≤ 0.01

*** P value ≤ 0.001

Overall these data suggested that similar inflammatory processes occurred in the thalamus during the early stages of infection regardless of the strain of scrapie used or the cellular pattern of PrPSc deposition. The timing of the upregulation of neuroinflammatory genes differed among the strains, but appeared to correlate with the pace of the infection in thalamus for each of the scrapie strains studied.

### Comparison of gene expression in scrapie versus two CNS viral models

The upregulation of neuroinflammatory genes in early scrapie appeared to follow the generation of PrPSc and the development of gliosis. The activated astroglia and microglia are likely to contribute to the profile of neuroinflammatory gene upregulation observed in our studies. Therefore, we next investigated whether the expression pattern of neuroinflammatory genes in scrapie was different from that seen in two fatal CNS viral diseases with prominent gliosis reactions: LaCrosse Virus (LACV), a bunyavirus causing encephalitis in children and mice [31–34] (reviewed in [35]), and BE virus, a chimeric neurovirulent retrovirus of mice [36, 37]. After ip inoculation in young mice, each of these viruses causes a fatal disease with prominent astrogliosis and microgliosis. In the case of BE virus neurons are not infected, and there is minimal degenerative pathology [37–39]. However, in LACV neurons are infected and killed, and in addition to gliosis there is a strong leukocyte infiltration from the periphery [31, 32, 34].

By analysis using qRT-PCR 26 genes noted to be elevated during scrapie were upregulated after infection with LACV (Table 4). Since many of these genes are known to be expressed by activated astroglia and/or microglia, the strong gliosis response seen with both scrapie and LACV might account for this overlap in their gene expression profiles. However, LACV infection also upregulated an additional 31 genes that were not elevated by scrapie (S2 Table). *Ccr2* was one of these latter genes, and its upregulation by LACV correlated with leukocyte infiltration in the LACV infected brain. This infiltration in turn might give rise to the very high expression levels of cytokine genes seen in LACV infection as well as the elevation of many genes not upregulated by scrapie (S2 Table).

**Table 4:** Number of neuroinflammatory genes increased in 22L scrapie-infected mice at 80 dpi compared to mice infected with LaCrosse virus (LACV) at 5 dpi.

Genes^1^	Increased in Scrapie	Not Increased in Scrapie
Increased in LACV	26	31
Not Increased in LACV	0	12

^1^Detailed data are shown in S2 Table.

In contrast to LACV, BE retrovirus infection induced upregulation of only 17 genes in the array tested, and 15 of these were also upregulated in scrapie (Table 5). Also, of the 27 genes upregulated in scrapie, eleven were not increased in BE (Table 5). *Cxcl11* and *Cxcl1* were the only genes upregulated by BE and not by scrapie in these samples (S3 Table). *Cxcl11* was in fact found previously to be elevated in the late stages of scrapie [20]. Thus, the expression profiles for BE and scrapie were similar, but there were also significant differences. In the case of BE, the reasons for these differences were not clear. However, the microglial response appeared to be weaker in BE infection than in scrapie infection, as measured by expression of *Gpr84* (microglial activation marker) (S3 Table), and this might account for the lower number of elevated genes after BE infection.

**Table 5:** Number of neuroinflammatory genes increased in 22L scrapie-infected mice at 80 dpi compared to mice infected with BE retrovirus at 21–28 dpi.

Genes^1^	Increased in Scrapie	Not Increased in Scrapie
Increased In BE	15	2
Not Increased in BE	11	34

^1^Detailed data are shown in S3 Table.

## Discussion

In the present work we studied the spread of PrPSc from the injection site in the striatum to several areas distant from this site. Microinjection into a small area made it possible to localize progression of infection over time. Previous studies by others indicated that spread of scrapie infection from the periphery and within the CNS was primarily via nerves using neuroanatomical pathways [40–45]. Here we compared three scrapie strains (22L, ME7 and RML). Outside the striatum, PrPSc from all three strains appeared first on the ipsilateral side in dorsomedial thalamus and lateral cortex [19], and then sequentially in contralateral cortex and ipsilateral substantia nigra (Fig 4). These findings supported the interpretation that the long distance spread for all three strains was via neural circuitry, and not by the brain interstitial fluid (ISF) as was seen previously over short distances within the striatum [18].

In the current paper, we studied early events of PrPSc infection in vivo by following the cellular associations of PrPSc at preclinical times (mostly 40–60 dpi). These early times were also selected in order to allow a precise identification of the cell types where PrPSc accumulated prior to the onset of extensive neuropathology. In pilot experiments, at the clinical end-point, PrPSc deposition usually became too dense for accurate determination of PrPSc accumulation around, in or near individual cells using dual staining. However, at earlier preclinical time-points using dual staining IHC in various brain regions, cell specificity of PrPSc accumulation was found to differ markedly among three scrapie strains. For example, starting at 40 dpi in thalamus and lateral cortex, 22L PrPSc accumulated mainly around parenchymal astroglia in all areas distant from the needle track including lateral cortex, thalamus, hypothalamus and substantia nigra. Other works previously found a similar possible association of 22L PrPSc with hippocampal astroglia at 56 dpi, but data on other brain regions was not reported [46]. In contrast to strain 22L, strain ME7 PrPSc almost never localized with astroglia, microglia or oligodendroglia, but instead associated primarily with neurons and neuropil, similar to what was previously reported at clinical times by others [12, 15].

Interestingly, strain RML appeared to combine the properties of strains 22L and ME7. For example, in thalamus and cortex, RML PrPSc colocalized mostly with astroglia, which was similar to 22L. However, in substantia nigra and hypothalamus, RML colocalized with both astroglia and neurons as well as neuropil, and similar findings were also noted in pons and vestibular nuclei. Our findings with RML were similar to studies using the closely related scrapie strains 79A and 79V, where PrPSc was associated with neurons and astroglia in several brain regions at clinical time-points [15]. Because of the early times of observation of these strain-specific cellular PrPSc accumulations in our experiments, these cellular associations are unlikely to be the result of extensive neuropathology; instead they more likely represent patterns of host/pathogen cellular interactions with PrPSc from individual scrapie strains.

The impact of the astroglial association of PrPSc of strains 22L and RML is not known. Possibly PrPSc accumulation of astroglia results in higher local levels of PrPSc, which in turn might increase the tempo of the disease. Indeed, 22L and RML have been found to progress more rapidly than strain ME7 by others [10] and by us (see methods for data on microinjections). Furthermore, astrocytic PrPSc has been previously shown to mediate neuronal damage indirectly by interaction with adjacent neuronal processes, even in the absence of PrPC expression on neurons [47].

The mechanism of association of PrPSc from specific scrapie strains with particular cell types is also not clear. Perhaps cell-specific molecules capable of acting as cofactors for strain-specific PrPSc amplification might be an explanation for these findings [48, 49]. Such molecules located on the external surface of the plasma membrane of specific cell types could potentiate PrPSc localization and new generation around neurons or astroglia. Similarly there might be intracellular factors capable of favoring intracellular PrPSc formation in specific cell types [50]. Neuropil PrPSc accumulation might be favored by factors on axons or dendrites or on glial cell processes located in these areas. If such factors could be identified in the future, this might provide new targets for drug therapy of specific strains of prion diseases. This same principle might also apply to other more prevalent neurodegenerative diseases where protein aggregation within or near specific cell types is a common feature.

In previous control experiments, the inflammatory gene upregulation associated with microinjection into the striatum was cleared within 14 days and was never detected in the thalamus. In the current study, using mRNA derived from thalamus at various early times starting at 40 days after infection, we were able to obtain data on expression levels of numerous transcripts possibly involved in the host response to scrapie infection and brain injury. By using quantitative RT-PCR arrays, the sensitivity for detection of transcripts was markedly increased above that seen in standard hybridization microarrays [20, 51–53]. This resulted in detection of numerous transcripts possibly overlooked previously. The three scrapie strains studied showed a slightly different temporal pattern of gene upregulation, as was also noted by IHC and Western blotting detection of PrPSc. The upregulated inflammatory genes were similar in the three strains correlating with early deposition of PrPSc and onset of glial activation.

Surprisingly, the scrapie strains did not show differing patterns of inflammatory gene upregulation, as may have been expected, due to the differing cell-type localizations of PrPSc observed by dual-staining IHC. However, neuronal injuries are well-known to stimulate neuroinflammation, and astrocytes might be more resistant to such damage. However, in the case of strain 22L with astroglial PrPSc, there may be additional PrPSc present on neurons at a level not detected by IHC. Alternatively, astrocytes injured by PrPSc accumulation might not take up glutamate properly leading to glutamate neurotoxic effects, or PrPSc accumulation on astrocytes might stimulate release of toxic molecules capable of injuring neurons [26, 54]. If so, neuroinflammation in all three scrapie strains may be stimulated primarily by neuronal damage induced directly or indirectly by PrPSc, and this might explain the similarity of the patterns of gene upregulation we observed with the three strains studied.

The chemokine ligands upregulated at early times from 40–60 dpi were: *Cxcl10*, *Ccl2*, *Ccl4*, *Ccl5*, *Ccl7*, and *Ccl8*. The receptors for these ligands are *Cxcr3*, *Ccr1*, *Ccr2*, *Ccr3*, and *Ccr5*. These systems are complex as most receptors can bind multiple ligands and each ligand can often bind multiple receptors [55]. Activation of these Ccr receptors leads to activation of many signaling pathways culminating in activation of *Erk*, *Jun*, *STAT*s, and *NF-kB*, which are known to be stimulated in the brain during scrapie infection [20, 56–60]. Several of these receptors have been individually assessed for their role in scrapie pathogenesis. Deletion of *Ccr1* (ligands: Ccl5, Ccl7, and Ccl8) led to a compensatory increase in *Ccr5* and *Ccl3* resulting in shortened survival times [56]. In contrast, elimination of either *Ccr2* (ligands: Ccl2, Ccl7, Ccl8, and Ccl12) or *Ccr5* (ligands: Ccl3, Ccl5, and Ccl8) did not alter prion incubation times [61]. Interestingly, elimination of *Cxcr3* (ligands: Cxcl9, Cxcl10, and Cxcl13) led to a 30 day increase in survival times but greater gliosis and PrPSc accumulation was noted [62]. The diversity of these effects makes definitive conclusions difficult, and considerable redundancy in these cytokine stimulatory systems may be confusing the outcome. Perhaps experiments using simultaneous deletion of multiple receptors will be required to better understand the roles of cytokines and their receptors in scrapie and other CNS diseases.

To understand whether neuroinflammatory gene expression differed in scrapie versus other brain diseases with similar levels of astrogliosis, we attempted to make cross-platform comparisons of neuroinflammatory gene upregulation between our scrapie data versus hybridization array data from traumatic brain injury and tauopathy models [63, 64]. However, these comparisons gave unsatisfactory results, probably because the qRT-PCR method used for scrapie was much more sensitive than the use of hybridization arrays. For such comparisons the gene expression methods should have equal sensitivity. For this reason, we compared the scrapie data with LACV and BE infections where we had data using the Super-Array qRT-PCR method. Scrapie, LACV and BE all provoked strong gliotic responses to infection, and in all three infections we found upregulation of multiple genes that probably were derived from activated astroglia and microglia. However, marked differences between scrapie and either LACV or BE were also noted. Most strikingly LACV had a strong increase in *Ccr2* correlating with leukocyte infiltration, which was not seen in scrapie or BE. There was also extensive neuronal death induced by LACV, which was not observed in BE or in scrapie at the early time-points studied in these experiments. These two differences probably accounted for the larger number of upregulated genes and very high fold change values seen with LACV.

Comparison of BE and scrapie also revealed significantly different profiles of neuroinflammatory gene expression. For example, of the 26 genes upregulated in scrapie only 15 were up in BE (Table 5). Furthermore, although activated microglia were seen in BE infected mice by IHC, based on the expression levels of microglial markers, *Gpr84* and *Aif1*, the number of activated microglia was less in BE than in scrapie. This was surprising because the BE mice used in this study were already beginning to have clinical signs and would have died in the next 5–6 days. Although microglia are a major target of BE virus infection in brain, possibly this infection might decrease the amount of microglial activation. In contrast, astroglial activation as assessed by IHC and by upregulation of *Gfap* and *Cxcl10* appeared to be strong in both scrapie and BE infection.

The comparison of neuroinflammatory gene profiles in three scrapie strains and in two other CNS viruses in this study supports the conclusion that reactive gliosis involving astroglia and microglia is likely more diverse than predicted by simply observing the activated glial cells by pathology and IHC. Each disease has its own individual gene profile that should ultimately provide extensive information about the processes taking place. However, our information about the detailed functions of many of the neuroinflammatory cytokines and their receptors is limited. The complexity of these ligands and their receptor specificity in the setting of the central nervous system makes this a challenging problem for the future.

## Materials and Methods

### Ethics statement

All mice were housed at the Rocky Mountain Laboratory (RML) in an AAALAC-accredited facility in compliance with guidelines provided by the Guide for the Care and Use of Laboratory Animals (Institute for Laboratory Animal Research Council). Experimentation followed RML Animal Care and Use Committee approved protocols 2011–04 and 2014–23.

### Scrapie infections

C57BL/10 (C57) mice were originally obtained from Jackson Laboratories and have been inbred at RML for several years. Young adult male mice, weighing 26–30 g were used for all stereotactic inoculations. In order to inject a high amount of infectivity while producing a minimum of damage from the volume of inoculum, mice were inoculated with 0.5 μl of 10% scrapie brain stock strains 22L, RML, and ME7 over 2 minutes using a pump as previously described [19, 65]. These stocks had been titered previously in C57 mice and contained the following ID_50_ in each 0.5 microliter volume: 22L, 1.0 x 10^5^; RML, 4.0 x 10^3^; ME7, 8.3 x 10^3^. Average times to clinical disease using this microinjection protocol were as follows: strain 22L, 154 dpi; strain RML, 149 dpi; strain ME7, 174 dpi. At selected time points post-inoculation, mice were euthanized by isoflurane anesthesia overdose followed by cervical dislocation. Brains were removed and immersed in 10% neutral buffered formalin (3.7% formaldehyde) for histology. For future use in western blot and RNA gene expression assays, brains were cut in half sagittally and the region of the thalamus was removed by careful gross dissection. To define the thalamus in the fresh brain the corpus callosum was used as the landmark for the dorsal border, the septum as the frontal border, a line vertical from the interpeduncular fossa as the caudal border and a final cut defining the ventral border was made between the estimated levels of the thalamus and hypothalamus. The resulting thalamic tissue (approximately 40 mg) was frozen in liquid nitrogen.

### Viral infections

For BE virus C57 mice could not be used as they are not susceptible to this virus or to many other murine retroviruses [36]. Therefore,129S1 mice were infected within 24 h of birth by intraperitoneal injection (i.p.) with 100 μl of cell culture supernatant containing 10^4^ focus-forming units of BE virus per mouse. BE-infected mice were observed daily and euthanized with the onset of clinical signs as described [66]. For LACV infections 3 week old C57BL/6 mice were inoculated i.p. with 10^3^ plaque-forming units of LACV human 1978 stock in phosphate-buffered saline (PBS) in a volume of 200 μl per mouse. LACV-infected mice were observed daily for signs of neurological disease and euthanized as described [67].

### Immunoblot detection of PrPSc

Dissected brain tissue from the thalamus was homogenized in PBS to create a 20% w/v brain homogenate (BH). Homogenization was done using a mini-bead beater homogenization system for 45 seconds on the homogenate setting. For proteinase K (PK) treatment, samples were incubated with detergents and PK as follows: 20 μl of a 20% tissue homogenate was adjusted to 100 mM Tris HCl (pH 8.3), 1% Triton X-100, and 1% sodium deoxycholate in a total volume of 31 μl. Samples were treated with 50 μg/ml of proteinase K (Roche Diagnostics) for 45 minutes at 37°C. The reaction was stopped by adding 2 μl of 100 mM Pefabloc (Roche Diagnostics) and placed on ice for 5 min. An equal volume of 2X Laemmli sample buffer (Biorad, Hercules, CA) was added, and then tubes were boiled 5 minutes. Samples were run immediately or frozen at -20°C until electrophoresed on a 16% Tris-Glycine SDS-PAGE gel (Life Technologies, CA) and blotted to PVDF using a 7 minute transfer, program 3 (P3) on an iBlot (Life Technologies, CA) device. Gels were transferred to polyvinylidene difluoride membranes using the iBlot transfer system (Life Technologies). Membranes were probed with a 1:100 dilution of monoclonal human anti-PrP antibody D13 derived from cell culture supernatants produced in our laboratory from CHO cells expressing the D13 antibody construct [19] that were kindly provided by Dr. R. Anthony Williamson. Monoclonal antibody D13 recognizes residues 94–105 in PrP [68] derived from mouse, hamster and squirrel monkey, and has been extensively used for detection of PrP in immunoblots and immunohistochemistry. The secondary antibody was peroxidase-conjugated anti-human IgG, used at 1:5,000 (Sigma), and immunoreactive bands were visualized using an ECL (Thermo Scientific) detection system. Densitometry on unsaturated immunoreactive bands was performed on exposed film using the Bio-Rad ChemiDoc MP system. Adjusted volumes for immunoreactive bands were calculated by taking the total band volume and subtracting the global background using Image Lab software version 5.0 (Bio-Rad).

### Immunohistochemical detection of PrPSc, neurons, microglia, and astroglia

Brains were removed and placed in 10% neutral buffered formalin for 3 to 5 days. Whole brains were divided coronally into 4 regions: (1) olfactory bulb to Bregma including the entire striatum, (2) middle thalamic area, (3) midbrain and (4) cerebellum. These tissues were then processed and embedded in paraffin. Sections were cut using a standard Leica microtome, placed on positively charged glass slides, and air-dried overnight at room temperature. The following day slides were heated in an oven at 60°C for 20 min. For all mice serial sections were obtained in the four regions mentioned above, and multiple sections were examined to confirm the reproducibility and regional extent of the pathology.

Deparaffinization, antigen retrieval and staining were performed using the Ventana automated Discovery XT stainer. Because of the intense aggregation of PrPSc immunostaining of PrPSc requires stringent antigen retrieval using high temperatures. In the present experiments PrPSc antigens were exposed by incubation in CC1 buffer (Ventana) containing Tris-Borate-EDTA, pH 8.0 for 100 minutes at 95°C as previously described [69]. Staining for PrP was done using human anti-PrP monoclonal antibody D13 described above. For immunohistochemistry, D13 culture fluid was used at a dilution of 1:100 for 2 hours at 37°C. The secondary antibody was biotinylated goat anti-human IgG at 1:250 dilution (Jackson ImmunoResearch, West Grove, PA), and streptavidin-biotin peroxidase was used with DAB as chromogen (DAB Map kit; Ventana Medical Systems, Tucson, AZ). Hematoxylin was used as a counterstain for all slides.

In order to study the localization of PrPSc with regard to specific brain cell types, slides were dual stained with D13 followed by one of several primary antibodies listed below. Slides were first stained with D13 using the complete protocol described above, finishing with DAB application. Then, without additional antigen retrieval, the second primary antibody was applied and staining was completed as described below, finishing with Fast Red application and hematoxylin counterstaining. The overall experimental plan and number of mice examined by pathology per group is shown in Table 6. In most cases 3 or more mice per time-point were examined, but occasionally when multiple time-points were examined only 2 mice were available. The goal in this study was to look at cell specificity of PrPSc accumulation early after spread to various brain regions. This required that PrPSc density be much less dense that was seen at the clinical end-point. Therefore, mice were often examined at different times in various brain regions, and we used time-points giving the clearest cell specificity of PrPSc accumulation. The photomicrographs shown in the figures are representative of typical fields seen in replicate mice. In previous single-staining experiments, comparing D13 versus humanized monoclonal antibody D18, which recognizes PrP residues 133–157 [68], we found very similar patterns of PrPSc deposition.

**Table 6:** Overview of number of mice per group in study of cellular accumulation of PrPSc by immunohistochemistry at various time-points after scrapie microinjection using three different scrapie strains.

Inoculum^a^	Mice^b^	20d^c^	40d	60d	80d	100d	clinical^d^
22L	C57	3^e^	6	2			4
ME7	C57	3	2	6	2	3	4
RML	C57	3	2	5	2		4
NBH	C57	3	3				

^*a*^ Inoculum was 0.5 μl of 10% scrapie brain homogenate (strains 22L, RML or ME7) or normal brain homogenate (NBH) microinjected into the striatum as described in the methods.

^*b*^ Mice were C57BL/10 (C57) as described in methods.

^*c*^ Time-points tested are given as days (d) post-injection.

^*d*^ Clinical mice used for pathology controls were infected with scrapie by intracerebral macroinjection of 30 microliters of a 1% scrapie brain homogenate into the left parietal lobe as previously described [19].

^*e*^ Number of mice per group.

The following primary antibodies were used in dual staining at the dilutions shown: Rabbit anti-Iba1 (1:2000) was a gift from Dr. John Portis, LPVD, RML, Hamilton, MT. Other antibodies were rabbit anti-glial fibrillary acidic protein (GFAP)(Dako #Z0334)(1:3500), rabbit anti-oligodendrocyte factor 2 (Olig2)(Millipore #9610)(1:50), rabbit anti-NeuN (Millipore #ABN78)(1:1000). Primary antibodies were diluted in PBS containing stabilizing protein and 0.1% Proclin 300 (Ventana Antibody Dilution Buffer). Diluent without antibody was used as a negative control. Ventana streptavidin-biotin alkaline phosphatase system (Red Map Kit, Ventana) was used to detect Iba1, GFAP, Olig2, and NeuN as described previously [70] with the exceptions that the secondary antibody was goat anti-rabbit Ig, (Biogenex, HK336-9R) and Fast Red chromogen was used. Additional dual staining was done with primary antibodies against GFAP with DAB chromogen followed by antibody against Olig2 with Fast Red chromogen. Slides were examined and photomicrographs were taken and observed using an Olympus BX51 microscope and Microsuite FIVE software. Discovery XT Staining Module software was used to design the dual stain procedures.

### RNA isolation

For mice infected with scrapie, thalamus tissue was excised and homogenized in 1.0 ml ZR RNA Buffer (Zymo Research) and stored up to 5 days at -80°C before processing. Total RNA was isolated using the Quick-RNA MiniPrep (Zymo Research), eluted with 75 ul nuclease-free water with 1 x RNase Inhibitor (SUPERase-In, Ambion), and stored at -80°C until use. For mice challenged with LACV or BE, brains were removed and total RNA isolated as previously described [66, 67].

### qRT-PCR analysis

For quantitative analysis of changes in transcription using qRT-PCR arrays, 400ng of high-quality RNA from each sample was reverse transcribed to synthesize cDNA using the RT^2^ First Stand Kit per manufacturer’s instructions (Qiagen). Each cDNA reaction was mixed with 2x RT2 SYBR Green Mastermix purchased from Qiagen with RNase-free water to a final volume of 1.3 ml. Ten microliters of the mixture was then added to each well of a 384-well format plate of the Mouse Inflammatory Cytokine & Receptors super array PAMM-011ZE (Qiagen). The analysis was carried out on an Applied Biosystems ViiA 7 Real-Time PCR System with a 384-well block using the following conditions: 1 cycle at 10 min, 95°C; 40 cycles at 15 s, 95°C then 1 min, 60°C with fluorescence data collection. Melting curves were generated at the end of the completed run to determine the quality of the reaction products. Raw threshold cycle (C_t_) data was collected with a C_T_ of 35 as the cutoff. C_T_ data was analyzed using the web-based RT² Profiler PCR Array Data Analysis (http://pcrdataanalysis.sabiosciences.com/pcr/arrayanalysis.php). All C_t_ values were normalized to the average of the Ct values for the housekeeping genes *Actb*, *Gapdh*, and *Hsp90ab1*. Changes in transcription were calculated by the software using the ΔΔC_T_ based method [71]. Statistical analysis was performed using the unpaired Student’s t-test to compare the replicate 2^(-ΔC^_T_^)^ values for each gene in the control group versus infected groups. A mean of ≥ 2.0 fold change and p-value of ≤ 0.05 considered significant. Each treatment and control group consisted of a minimum of 3 independent RNA samples.

To determine changes in *Aif1* (encoding IBA1), *Il12b* (encoding IL12p40), *Cx3cr1*, *Vim* (encoding Vimentin), *Gpr84*, and *Gfap* transcription during disease by qRT-PCR, 200 ng of RNA was reverse transcribed into cDNA using the RT^2^ Easy First Stand kit (Qiagen) as indicated by the manufacturer. The cDNA product was diluted 1:2 with nuclease-free water and stored at -20°C until use. Twenty-five microliter reactions were performed in a 384 well format using 12.5 ul RT^2^ qPCR Mastermix (Qiagen), 10.5 nuclease-free water, 1.0 diluted cDNA template, and 1.0 ul Primer Assay Mix. qRT-PCR conditions were as follows: 1 cycle at 10 min, 95°C; 40 cycles at 15 s, 95°C then 1 min, 60°C with fluorescence data collection. Melting curves were generated at the end of the completed run to determine the quality of the reaction products. Raw threshold cycle (C_T_) data was collected with a C_T_ of 35 as the cutoff. The results were calculated using the ΔΔC_T_ method as above, where relative amounts of RNA were normalized to the geometric mean (C_T_) of Gapdh and Actin. Primer Assay Mixes for *Aif1*, *Il12b*, *Cx3cr1*, *Vim*, *Gfap*, *Gapdh*, and *Act* were purchased from Qiagen. Specific primer sequences for *Gpr84* were CTGACTGCCCCTCAAAAGAC as the forward primer and GGAGAAGTTGGCATCTGAGC as the reverse primer.

## Supporting Information

S1 Table Alphabetical list of the eighty-six neuroinflammatory genes assessed by qRT-PCR. (PDF)

S2 Table Mouse inflammatory gene expression profiles during LaCrosse virus (LACV) versus 22L scrapie infection relative to uninfected mice. (PDF)

S3 Table Mouse inflammatory gene expression profiles during BE retrovirus versus 22L scrapie infection relative to uninfected mice. (PDF)
