# Supplementary material for: Prion Strain Differences in Accumulation of PrPSc on Neurons and Glia Are Associated with Similar Expression Profiles of Neuroinflammatory Genes: Comparison of Three Prion Strains
Source: PLoS Pathog. 2016 Apr 5;12(4):e1005551. doi: 10.1371/journal.ppat.1005551 (PMC4821575; doi:10.1371/journal.ppat.1005551)
Supplement: S2 Table — (PDF) [file ppat.1005551.s002.pdf]

S2 Table. Mouse inflammatory gene expression profiles during LaCrosse virus (LACV) versus 22L scrapie infection relative to uninfected mice.

Genes not increased in LACV or scrapie		Genes increased in LACV only			Genes increased in scrapie and LACV			
**Gene**		**Gene**	**FC LACV 5 dpi**		**Gene**	**FC LACV 5 dpi**		**FC 22L 80 dpi**
Bmp2		*Cxcl11*	249.0***		*Cxcl10*	1243.3***		78.9***
Ccl17		*Cxcl1*	240.5***		*Ccl4*	259.6***		31.5***
Ccr10		*Ifng*	229.1***		*Il12b*	284.0***		30.6***
Cx3cl1		*Cd40lg*	67.6***		*Cxcl9*	2504.0***		26.4**
Cxcl12		*Ccl20*	45.6***		*Cxcl13*	136.2***		23.9***
Fasl		*Il3*	29.9**		*Ccl5*	209.4***		18.7***
Il16		*Ccr4*	27.1***		*Ccl2*	1782.9***		15.3***
Il6st		*Ccr2*	24.4***		*Tnf*	512.0***		15.2***
Mif		*Cxcr5*	21.3***		*Ccl12*	187.4***		13.4***
Pf4		*Il13*	21.0***		*Gfap*	9.0***		10.9***
Tnfrsf11b		*Cxcl15*	18.9***		*Ccl8*	62.7***		9.9***
Vegfa		*Tnfsf10*	18.4***		*Il1b*	14.7***		8.8***
		Il4	11.3***		*Ccl3*	179.8***		7.9***
		Ccl19	10.4***		*Ccl9*	8.5***		6.5***
		Il11	10.1***		*Ccl6*	10.6***		6.3***
		Ccr5	8.9***		*Ccl7*	512.0***		6.3***
		Ccl22	7.1**		*Il1a*	16.0***		4.2***
		Il6ra	7.0***		*Il2rg*	24.6***		3.5***
		Il5ra	6.4***		*Gpr84*	23.1***		3.3***
		Il15	5.4***		*Ccr3*	12.6***		3.0***
		Ccl24	5.3*		*Il2rb*	47.5***		3.0**
		Ccr8	5.0**		*Cxcr3*	16.4***		2.9*
		Ltb	4.4**		*Ccr1*	34.1***		2.8***
		Lta	4.2*		*Cxcl5*	2.3**		2.4**
		Ccr6	3.7**		*Ccl11*	75.1***		2.3**
		Ccl1	2.8***		*Il10ra*	6.3***		2.2***
		Il17b	2.8***					
		Spp1	2.7**					
		Il1r1	2.6***					
		Spp1	2.5**					
		Cx3cr1	2.1***					
		Il10rb	2.1***					

FC = Fold Change in expression in infected mice vs. uninfected control mice. 69 genes analyzed.
* P value ≤ 0.05, ** P value ≤ 0.01, *** P value ≤ 0.001
No genes were increased in scrapie only.
