# Supplementary material for: Prion Strain Differences in Accumulation of PrPSc on Neurons and Glia Are Associated with Similar Expression Profiles of Neuroinflammatory Genes: Comparison of Three Prion Strains
Source: PLoS Pathog. 2016 Apr 5;12(4):e1005551. doi: 10.1371/journal.ppat.1005551 (PMC4821575; doi:10.1371/journal.ppat.1005551)
Supplement: S3 Table — (PDF) [file ppat.1005551.s003.pdf]

S3 Table. Mouse inflammatory gene expression profiles during BE retrovirus versus 22L scrapie infection relative to uninfected mice.

Genes not increased in BE or scrapie		Genes increased in BE only			Genes increased in scrapie and BE					Genes increased in scrapie only
**Gene**		**Gene**	**FC BE 21-28 dpi**		**Gene**	**FC BE 21-28 dpi**		**FC 22L 80 dpi**		**Gene**
Ccl1		*Cxcl11*	5.3***		*Cxcl10*	149.1***		78.9***		*Ccl9*
Ccl17		*Cxcl1*	3.6***		*Ccl4*	11.9***		31.5***		*Ccl6*
Ccl19					*Cxcl9*	45.9***		26.4**		*Il2rg*
Ccl22					*Cxcl13*	52.3***		23.9***		*Aif1*
Ccr10					*Ccl5*	13.9***		18.7***		*Gpr84*
Ccr2					*Ccl2*	14.4***		15.3***		*Ccr3*
Ccr4					*Tnf*	6.5*		15.2***		*Il2rb*
Ccr5					*Ccl12*	14.6***		13.4***		*Cxcr3*
Ccr6					*Gfap*	4.2***		10.9***		*Ccr1*
Ccr8					*Ccl8*	9.5**		9.9***		*Cxcl5*
Cd40lg					*Il1b*	2.6***		8.8***		*Il10ra*
Csf2					*Ccl3*	7.3***		7.9***		
Csf3					*Ccl7*	7.1***		6.3***		
Cx3cl1					*Il1a*	5.7**		4.2***		
Cxcl12					*Ccl11*	4.2**		2.3**		
Cxcl15										
Cxcr5										
Ifng										
Il10rb										
Il11										
Il13										
Il15										
Il16										
Il17b										
Il1r1										
Il4										
Il5ra										
Il6ra										
Il6st										
Lta										
Ltb										
Mif										
Pf4										
Spp1										

FC = Fold Change in expression in infected vs. uninfected control mice. 62 total genes analyzed.
* P value ≤ 0.05, ** P value ≤ 0.01, *** P value ≤ 0.001
